# The influence of simulated patient counsellingtraining sessions on intern communication skills: A questionnaire based cross sectional study

**DOI:** 10.6026/973206300222853

**Published:** 2026-05-31

**Authors:** Puneeta Gupta, Neerupama Dogra, Rohit Raina, Supinder Singh, Sunakshi Sharma

**Affiliations:** 1 Department of General Medicine, Acharaya Shri Chander College of Medical Sciences (ASCOMS), Jammu, India; 2 Department of Ophthalmology, Acharaya Shri Chander College of Medical Sciences (ASCOMS), Jammu, India; 3 Department of General Medicine, AIIMS, Bathinda, Punjab, India

**Keywords:** Communication skill, role play, simulation, Kalamazoo consensus statement

## Abstract

The poor communication skills among doctors are one of the reasons for workplace violence (WPV) against medical professionals. The usefulness of role play as a tool to teach basic communication and counseling skills to the interns (who would very soon be first year residents) and to analyse and assess the perception of students and faculty towards role play sessions as a teaching-learning method. The 36 interns posted in the department of medicine were randomly divided into 6 groups and distributed as a treating physician, patient, family member and observers and were asked to perform role play of given clinical scenarios before and after the workshop conducted in the department. The pre-training average score for each response had lower values (M = 5, SD = 0.9) than the post-training average score for each response (M =8.46, SD = 1.92). Role-play sensitization workshop followed by detailed debriefing had a positive impact on communication skills in interns in clinical based scenarios.

## Background:

Communication skills in medicine don't merely consist of obtaining good history and collecting relevant information about the illness, from the patient and the family members, but also sharing their anxiety and emotional turmoil (empathy) and to be able to build a relationship with the them based on understanding and respect for patient's expectations and perceptions, so that there is no hesitation on both sides when it comes to implementing difficult treatment decisions [1, 2]. It has been observed that the good doctor-patient communication impacts a range of patient outcomes in a positive way as it enhances patient empowerment resulting in better compliance and adherence with treatment and decreased chances of medical error in addition to encouraging healthy behaviour among patients in both acute and long-term care [2, 3, 4, 5]. Moreover, the poor communication skills among doctors can be one of the reasons responsible for workplace violence (WPV) against medical professionals [6]. Different studies have reported WPV in the last few years in various hospitals across India to vary from 35.7% to 78% [6]. Despite the awareness about the crucial role of communications in healthcare, this aspect, however, has not been given much importance in the teaching curriculum, resulting in young doctors entering the clinical practice with inadequate training [6, 7, 8, 9, 10, 11, 12]. Therefore, medical faculties all over the world are increasing efforts to promote the learning of communication skills by medical students [12]. Therefore, it is of interest to evaluate the usefulness of role play as a tool to teach basic communication and counselling skills to the interns who would very soon be first year residents and the perceptions of students and faculty on using role play to teach counselling and communication skills.

## Materials and Methods:

The ethical clearance was obtained from the Institutional Ethics Committee for the proposed prospective and interventional study to be conducted over a period of six month. A workshop was organized in the department of medicine along with MEU (Medical Education Unit) members to sensitize the faculty to the concept and various aspects of role play. Afterwards four modules were designed by the faculty, finalized and validated after discussion within the MEU. Each module had one commonly encountered clinical problem in internal medicine as a task for role play sessions to assess communication skills. These included, counselling the patient's family about the diagnosis, treatment options and prognosis of the condition in a case of life-threatening haematemesis; counselling the patient of end stage renal failure for the need of initiation of maintenance dialysis; counselling a family of patient of COPD with respiratory failure with different treatment options, expected response to treatment and need for mechanical ventilation; counselling the family members of the patient recently diagnosed with malignancy of lungs and possible treatment options including the palliative care. The "Calgary-Cambridge Guide to the Medical Interview" and "Kalamazoo Essential Elements Communication Checklist" was considered as the reference for preparing the module for the students. A didactic lecture highlighting the salient aspects of communications in clinical medicine was attended by all 36 interns participating in the study. As students under the new CBME curriculum are already sensitized to the concept of role play since first professional year, they were asked to perform the role play amongst themselves, (after attending the lecture on the importance of good communication skills) to see how they will interact with patients in some of the common conditions seen in the OPDs, wards and emergency rooms, in presence of the designated faculty members. The interns were divided into 6 groups and were allocated randomly the roles of treating doctor, patient and family member and also the observers within each group. The performance was evaluated by the designated faculty based on the Kalamazoo consensus statement, recorded as pre-workshop score. Two days after this activity, a detail workshop explaining the various aspects of role play including the concept of debriefing, feedback and self-reflection, followed by a question-answer session, was conducted in the department of Medicine. The session also included a role-playing demonstration video which had both correct and incorrect ways to communicate with patient and the family members. After the sensitization workshop, the students were given one week for preparation of the role-play sessions and were asked to repeat same clinical scenarios, they had been asked to discuss with simulated patients, prior to workshop. The script of role play was in Hindi and each group was given 20-25 mins to conduct the session. All the sessions were facilitated be senior faculty member. At the end of the role play, there was a debriefing session stressing the importance of good communication skills as formulated in the module. The performance of the simulated doctors in each group was evaluated using the Kalamazoo Essential Elements Communication Checklist by the same faculty members (post workshop score), who evaluated them before the workshop. The participating interns and the faculty completed a questionnaire at the end of the session.

## Results:

**Figure 1:**
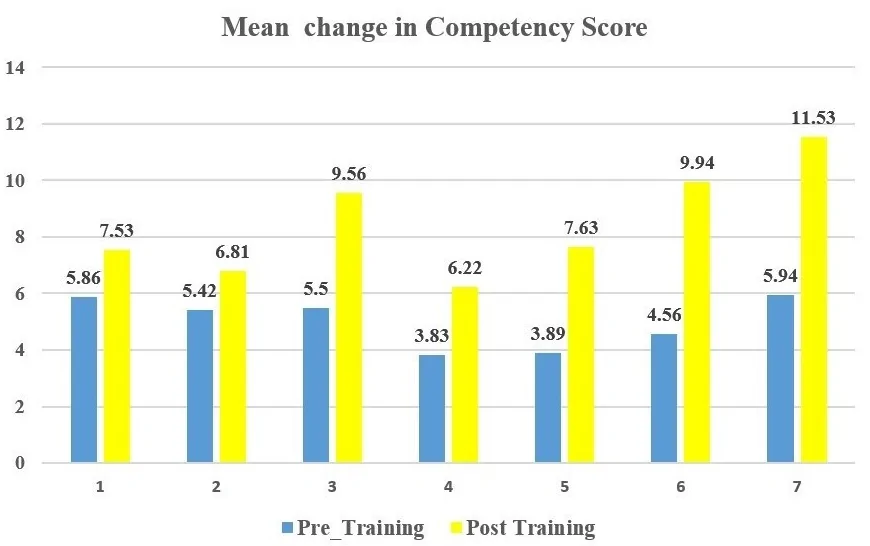
Bar Chart representing the Pre-training average score and post-test average score for each Response. Pre-training average score had lower values (M = 5, SD = 0.9) than the post-training average score for each response (M = 8.46, SD = 1.92). A t-test for paired samples showed that this difference was statistically significant, t (6) = -5.39, p = 0.002, 95% Confidence interval (-5.03, -1.89).

**Figure 2:**
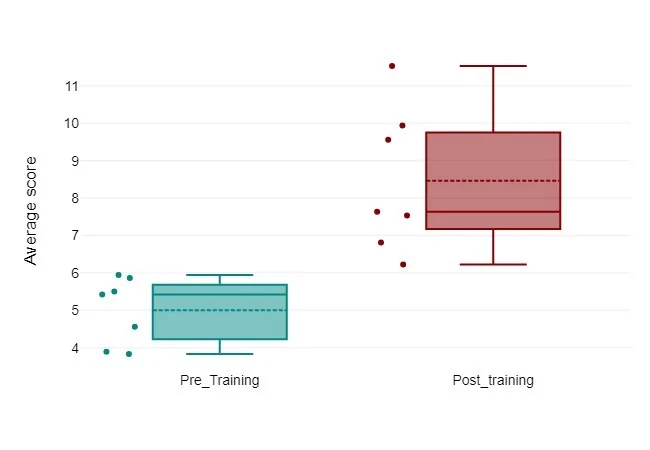
Box Whisker Plot representing the Pre-training average score and post-test average score for each Response. Pre-training average score had lower values (M = 5, SD = 0.9) than the post-training average score for each response (M = 8.46, SD = 1.92). A t-test for paired samples showed that this difference was statistically significant, t (6) = -5.39, p = 0.002, 95% Confidence interval (-5.03, -1.89).

**Figure 3:**
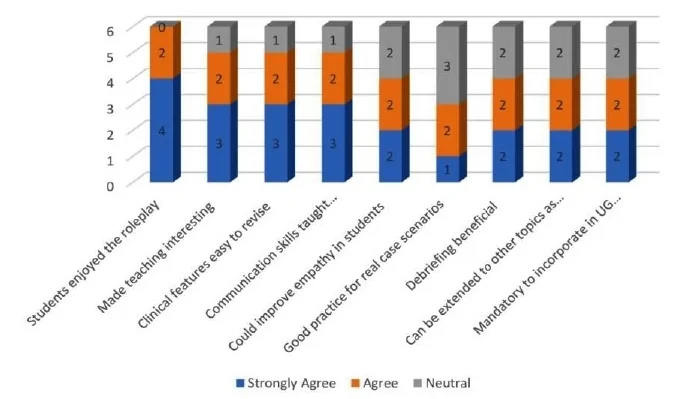
Bar chart representing the perception among teachers regarding role play.

The performance of simulated doctors in each group was assessed using the Kalamazoo Essential Elements Communication Checklist, with evaluations conducted by the same faculty members who performed the pre-workshop assessments. Upon completion of the workshop, both interns and faculty participants responded to a structured questionnaire. The components of the Kalamazoo Consensus Statement, along with the corresponding pre- and post-workshop scores, are presented in Table 1. Paired t-test is used to check the statistical significance and Shapiro Wilk and Q-Q plot was used to access the normality of the data. The Pre-training average score for each response had lower values (M = 5, SD = 0.9) than the post-training average score for each response (M = 8.46, SD = 1.92). A t-test for paired samples showed that this difference was statistically significant, t (6) = -5.39, p =0 .002, 95% Confidence interval (-5.03, -1.89) as illustrated in Figure 1 and Figure 2. All statistical analysis is done by using SPSS statistical software version 21. Graphs are prepared in excel and data tab studio. All 36 interns and six consultants from the faculty participated in pre and post workshop sessions and all of them filled the feedback questionnaire. Regarding the perception of the students about the use of role play as an effective communication tool 83.33% (30 out of 36) strongly agreed or agreed (SA+A) that session of role play as a teaching learning method was lively and interesting whereas 11.11% students didn't offer any opinion, neutral (4 out of 36) and 5.56% disagreed with the observation (2 out of 36) (Table 2). 83.33 % thought it made teaching more interesting as clinical features could be easily revised and they were able to teach more objectively. 66.67% of faculty strongly agreed or agreed that these sessions of role play on communication skills could improve empathy in students, debriefing sessions were very helpful and these sessions could be used in other departments as well and role play should be made mandatory for teaching communication skills in undergraduate curriculum (Table 3, Figure 3).

## Discussion:

Despite the awareness about the crucial role of communication in healthcare, this aspect has not been given enough importance in the teaching curriculum, resulting in young doctors entering the clinical practice with inadequate communication skills. The famous Canadian physician, Sir William Osler once stated that "A good physician treats the disease and a great physician treats the patient who has the disease." The various studies, in last few years, have addressed the importance of learning communication skills and have suggested and evaluated various different teaching-learning methods to effectively impart these soft skills to the future healers. Internship is a particularly interesting phase in medical schools, as students are participating in day to day management of the patients and are preparing themselves for their future role as first year post graduate students. Thus, it is an appropriate time for them to learn diverse aspects of patient management by both observing their seniors (hidden curriculum) and also by taking active part in their own learning. The simulation-based training is rapidly expanding as a core educational strategy for learning professional skills in healthcare, including communication abilities [6]. Simulation is a generic term that refers to an artificial representation of a real world situation aiming to achieve learning objectives through hypothetical situations (as against direct bedside teaching involving real patients) based on real clinical problems. Both terms "simulated patient" (SimP) and "standardized patient" are used interchangeably and both refer to programs in which the role of the patient is played by a trained person or by a professional (an actor) or an untrained person. By contrast, the term "peer role-play" typically refers to those settings in which students also play the role of the patient. In simulation-based training, the role-play sessions are planned to enact and rehearse various clinical situations to improve learner's proficiency and capability to face similar situations in real clinical practice. The role play sessions can be conducted repeatedly till the student feel confident to handle the sensitive and emotional issues as it is done in a controlled environment and there is no risk of annoying or hurting the patient (which is difficult to achieve in bedside teaching with real patients). The review of literature reveals that the various modalities are being used to train students for communication skills, e.g., videos, discussions, reflection, lectures, feedback sessions, vignettes and brainstorming sessions etc., The inclusion of role-play (simulation) in teaching methods has many benefits as it enables the learner to experience the problems he or she will face in the real world and actively apply the knowledge in simulated situations. The learner has a chance to experience the perspective of both partners in communication - the client and the caregiver, but in a controlled environment. Luttenberger et al. in their study pointed out the effectiveness of role play in imparting communication skills among second- year medical undergraduates [13]. Lavanya et al. and Nair in their study found that most students considered role play as a preferred tool to acquire proper communication skills [14, 15]. Manzoor et al. recommended that by engaging students in role plays, the components of both cognitive as well as the affective domain of medical education can be addressed [16]. In addition, role play offers the advantages of fostering affective skill development and empathy toward patients, thus warranting its inclusion in the medical curriculum [17, 18, 19]. Many other authors have reported that students had positive perceptions about role playing because it simulated real life experience into the classroom or training sessions. Our study demonstrated a significant difference in post-workshop scores compared with pre- workshop scores as interns performed better in all the components of the Kalamazoo consensus statements after the intervention. Our observations were similar to those seen in other studies done by other investigators [20, 21, 22]. The feedback from the students and faculty about the activity revealed that the detailed workshop helped to understand the need for communication in general and prepared them for their future role as a clinician in a non-threatening situation, which could not have been possible in didactic lecture and bedside teaching [23, 24, 25]. The results showed that students preferred role play sessions over case-based learning, tutorials, small group discussions and lectures as the most preferred learning tool to teach communication skills.

## Conclusion:

We show that role-play is an effective method for teaching communication skills which provide students with a no-risk platform for rehearsal and improvisation. Moreover, students also get the opportunity to work on their deficiencies and improve their skills, based on the feedback received from their peers and the faculty. Further, the faculty can come up with well-scripted clinical interaction of different case scenarios, which can be used as teaching-learning method in undergraduate and post-graduate teaching.

## Limitations:

Apart from the small sample size, few clinical scenarios were taken up for role play. There could have been an element of response bias as the students knew the researcher and consciously, or subconsciously, gave the response (that they thought), the interviewer wanted to hear. Some investigators have opined that in similar situations, some medical students reported feeling uncomfortable by all the attention and comparison and evaluation of their professional and unprofessional behaviors by the medical faculty and peers. The long-term impact of peer role play sessions in bringing around behavioral changes needs further investigation.

## Financial support and sponsorship:

Nil

## Declaration:

Authors declare that the manuscript is not submitted elsewhere and no pre-prints are published elsewhere.

## Figures and Tables

**Table 1:** Components of Kalamazoo consensus statement and scores pre and post workshop

**S. No.**	**Competency**	**Possible maximum Score**	**Mean Score before Training**	**Mean Score after Training**
**1**	**BUILDS RELATIONSHIP**			
	1.Greets and shows interest in patient as a person			
	2.Uses words that show care and concern throughout the interview	20	5.86	7.53
	3.Uses tone, pace, eye contact and posture that show care and concern			
	4.Responds explicitly to patient's statements about ideas and feelings			
**2**	**OPEN THE DISCUSSION**			
	1.Allows patient to complete opening statement without interruption	15	5.42	6.81
	2.Asks "Is there anything else?" to elicit full set of concerns			
	3.Explains and/or negotiates an agenda for the visit			
**3**	**GATHERS INFORMATION**			
	1.Begins with patient's story using open-ended questions (e.g. "tell me about")	20	5.5	9.56
	2.Clarifies details as necessary with more specific or "yes/no" questions			
	3.Summarizes and gives patient opportunity to correct or add information			
	4.Transitions effectively to additional questions			
**4**	**UNDERSTAND PATIENT'S PERSPECTIVE**			
	1.Asks about life events, circumstances, other people that might affect health	10	3.83	6.22
	2.Elicits patient's beliefs, concerns and expectations about illness and treatment			
**5**	**SHARES INFORMATION**			
	1.Assesses patient's understanding of problem and desire for more information	15	3.89	7.67
	2.Explains using words that patient can understand			
	3.Asks if patient has any questions			
**6**	**REACHES AGREEMENT**			
	1.Includes patient in choices and decisions to the extent s/he desires			
	2.Checks for mutual understanding of diagnostic and/or treatment plans	20	4.56	9.94
	3.Asks about patient's ability to follow diagnostic and/or treatment plans			
	4.Identifies additional resources as appropriate			
**7**	**PROVIDES CLOSURE**			
	1.Asks if patient has questions, concerns or other issues			
	2.Summarizes	25	5.94	11.53
	3.Clarifies follow-up or contact arrangements			
	4.Provides appropriate contact information if interim questions arise			
	5.Acknowledges patient and closes interview			

**Table 2:** Perception of students about using role play in teaching communication skills

**Questions**	**Strongly Agree (SA)**	**Agree (A)**	**Total% (SA+A)**	**Neutral**	**Disagree**	**Strongly Disagree**	**Total**
Whole session was engaging and interesting	28	2	28+2	4	2	0	36
Helped learning communication skills in a better way	24	4	24+4	4	4	0	36
Attitude towards patients improved	28	3	28+3	5	0	0	36
Easier transition to ward cases	20	8	20+8	8	0	0	36
Revision of Clinical features taught in lectures	20	4	20+4	12	0	0	36
Can be extended to other departments	16	6	16+6	10	4	0	36
Debriefing beneficial	29	2	29+2	5	0	0	36

**Table 3:** perception among teachers regarding role play

**Questions**	**Strongly Agree (SA)**	**Agree (A)**	**Total (SA+A)**	**Neutral**	**Total**
Students enjoyed the role play	4	2	6	0	6
Made Teaching interesting	3	2	5	1	6
Clinical features easy to revise	3	2	5	1	6
Communication skills taught more objectivity	3	2	5	1	6
Could improve empathy in students	2	2	4	2	6
Good practice for real case scenarios	1	2	3	3	6
Debriefing beneficial	2	2	4	2	6
Can be extended to other topics as well as other departments	2	2	4	2	6
Mandatory to incorporate in UG teaching	2	2	4	2	6
